# lncRNA Mirt2 Is Downregulated in Ulcerative Colitis and Regulates IL-22 Expression and Apoptosis in Colonic Epithelial Cells

**DOI:** 10.1155/2019/8154692

**Published:** 2019-10-07

**Authors:** Guangrong Ding, Yuzheng Ming, Yuling Zhang

**Affiliations:** Department of Gastroenterology, The Second People's Hospital of Lanzhou, Lanzhou City, Gansu Province 730046, China

## Abstract

lncRNA Mirt2 is a lipopolysaccharide- (LPS-) inducible inflammation inhibitor. We found that Mirt2 was downregulated in plasma of ulcerative colitis (UC) patients and the downregulation of Mirt2 distinguished UC patients from healthy controls. IL-22 was also downregulated in UC patients and positively correlated with Mirt2. Mirt2 overexpression led to upregulated, while Mirt2 siRNA silencing led to inhibited secretion of IL-22 from colonic epithelial cells treated with LPS. In addition, under LPS treatment, Mirt2 overexpression led to decreased, while Mirt2 siRNA silencing led to increased apoptotic rate of colonic epithelial cells. Therefore, Mirt2 is downregulated in ulcerative colitis and regulates IL-22 expression in colonic epithelial cells.

## 1. Introduction

In clinical practices, a common group of gastrointestinal disorders are inflammatory bowel diseases (IBD) [1]. Ulcerative colitis (UC) is one of the two major subtypes of IBD (the other subtype is Crohn's disease) [1]. UC causes unacceptable high morbidity rate [2]. Inflammatory response caused by foreign antigens and intestinal microbes is closely related to the occurrence of UC [3, 4], while the etiology of IBD is still hardly known. Proinflammatory cytokine- (such as TNF, IFN-*γ*, IL-17A/F, or IL-21) targeted therapies have been widely used in the clinical treatment of UC [5]. These therapies mainly focus on cytotoxicity reduction and inflammation inhibition [5, 6]. Even disease conditions can be controlled in most cases; further improvement in the clinical treatment outcomes is still needed to improve the recovery.

It is clear that some inflammatory mediators, such as IL-23, TNF-*α*, and IL-12, participate in the pathogenesis of UC, and inhibition of the production and secretion of such mediators is considered to be beneficial for disease control and recovery [7, 8]. Another possible therapeutic approach is to increase the secretion of certain anti-inflammatory cytokines, such as IL-22, which can inhibit intestinal inflammation [9]. Long (>200 nt) noncoding RNAs (lncRNA) are RNA transcripts with limited protein coding capacity but with regulatory function in gene expression [10]. lncRNA Mirt2 is a lipopolysaccharide- (LPS-) inducible inflammation inhibitor [11]. Therefore, we speculate that the inflammation inhibitory effects of Mirt2 may also be useful for the treatment of UC. Therefore, we investigated the role of Mirt2 in UC.

## 2. Materials and Methods

### 2.1. Research Subjects

To analyze the differential expression of Mirt2 in UC, this study included 68 patients diagnosed with UC and 68 healthy controls in the Second People's Hospital of Lanzhou during the time period from March 2016 to March 2018. Inclusion criteria of patients are as follows: (1) UC patients with the first diagnosis and (2) no therapies received before this study. Exclusion criteria are as follows: (1) patients transferred from other hospitals and (2) patients who were diagnosed with multiple clinical disorders. Healthy controls were selected to match the gender and age distributions of the patient group. All patients and controls were informed with the experimental principle, and this study was approved by the Ethics Committee of the aforementioned hospital before the admission of patients.

### 2.2. Plasma and Cells

Fasting blood (5 ml) was extracted from each participant before the initiation of any therapies. Blood was transferred to EDTA tubes and was centrifuged for 15 min at 1200 g to separate serum.

Human colonic epithelial cells (HCnEpC, Cell Applications, USA) were used. HCnEpCs were cultivated under the instructions provided by Cell Applications. To establish a UC cell model, LPS at a dose of 4 *μ*g/ml was used to treat HCnEpCs for 24 h before use.

### 2.3. Transient Transfection

A Mirt2 expression vector was constructed by Sangon (Shanghai, China) using the pcDNA3 vector. Mirt2 siRNA and negative control siRNA were designed and synthesized by GenePharma (Shanghai, China). Transient transfections were performed using Lipofectamine® 2000 Reagent (Sigma-Aldrich, USA) to transfect 10 nM Mirt2 expression vector, 10 nM empty vector (negative control, NC), 35 nM Mirt2 siRNA, or 35 nM negative control siRNA (negative control, NC) into 10^5^ HCnEpCs. Control (C) cells were cells without any transfections. Subsequent experiments were performed using HCnEpCs collected at 24 h after transfections.

### 2.4. RT-qPCR

HCnEpCs (10^5^) or plasma (0.2 ml) was mixed with 1 ml RiboZol™ RNA Extraction Reagent (VWR, USA) to extract total RNA. The SensiFAST™ cDNA Synthesis Kit (Bioline, USA) was used to perform reverse transcriptions after RNA samples were treated with DNase I. After that, qPCR reaction mixtures were prepared using SYBR Green Master Mix (Bio-Rad, USA) with 18S rRNA as endogenous control to analyze the expression of Mirt2. Three replicates were set for each reaction. Data analysis was performed using the 2^-ΔΔCT^ method.

### 2.5. ELISA

The Human IL-22 Quantikine ELISA Kit (D2200, R&D Systems, USA) was used to detect IL-22 in plasma and cell culture medium of HCnEpCs. Levels of IL-22 in plasma were normalized to pg/ml.

### 2.6. Cell Apoptosis Assay

HCnEpCs were collected at 24 h after transfections, followed by incubation with LPS at a dose of 4 *μ*g/ml. HCnEpCs were then washed twice with PBS. And then, binding buffer (500 *μ*l), PI solution (5 *μ*l), and FITC-labeled Annexin V (5 *μ*l) were used to mix with 10^6^ HCnEpCs, followed by incubation for 10 min in the dark. Finally, the FACSCalibur flow cytometer (BD, Lake Franklin, New Jersey, USA) was used to detect apoptotic cells.

### 2.7. Statistical Process

Three biological replicates were set for each experiment. Mean value was used to represent the data of 3 replicates. The unpaired *t*-test was used to test the differences between two groups of participants. One-way ANOVA and the Tukey test were used to test the differences among different cell treatment groups. Diagnostic analysis was performed using ROC curve analysis. Linear regression was used for correlation analysis. *p* < 0.05 indicated a difference with statistical significance.

## 3. Results

### 3.1. Plasma Mirt2 Was Downregulated in UC and Showed Diagnostic Values

**Figure 1:**
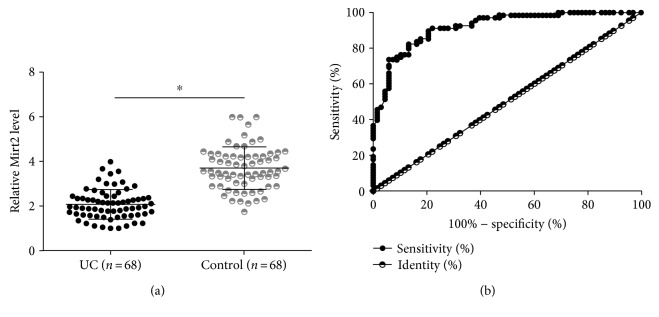
Plasma Mirt2 was downregulated in UC and showed diagnostic values. Mirt2 in plasma of the patient (*n* = 68) and control (*n* = 68) groups was detected by performing RT-qPCR, followed by data analysis using the unpaired *t*-test. Mirt2 levels in plasma were significantly lower in the patient group than in the control group (a), (^∗^*p* < 0.05). ROC curve analysis showed that downregulation of Mirt2 in plasma of UC patients distinguished UC patients from healthy controls (b).

It was observed that Mirt2 levels in plasma were significantly lower in the patient group than in the control group (Figure 1(a), *p* < 0.05). ROC curve analysis was performed to analyze the diagnostic values of Mirt2 for UC. In this analysis, UC patients were true positive cases and healthy controls were true negative cases. As shown in Figure 1(b), the area under the curve was 0.92, with a standard error of 0.023 and a 95% confidence interval of 0.87-0.96.

### 3.2. IL-22 Was Positively Correlated with Mirt2 in UC Patients

**Figure 2:**
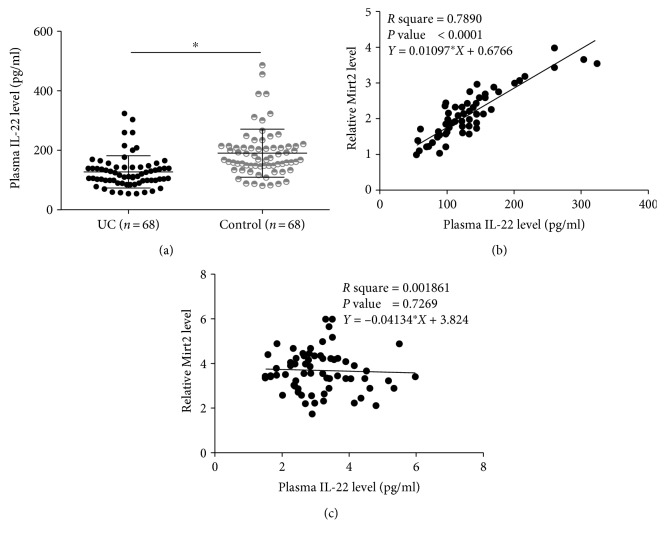
IL-22 was positively correlated with Mirt2 in UC patients. IL-22 in plasma of the patient (*n* = 68) and control (*n* = 68) groups was detected by performing ELISA, followed by data analysis by performing the unpaired *t*-test. Levels of IL-22 in plasma were significantly lower in the patient group than in the control group (a) (^∗^*p* < 0.05). Linear regression showed that IL-22 and Mirt2 were positively and significantly correlated in UC patients (b). However, the correlation between IL-22 and Mirt2 was not significant in UC patients (c).

Analysis of ELISA data showed that levels of IL-22 in plasma were significantly lower in the patient group than in the control group (Figure 2(a), *p* < 0.05). Correlations between IL-22 and Mirt2 were analyzed by linear regression. It was observed that IL-22 and Mirt2 were positively and significantly correlated in UC patients (Figure 2(b)). However, the correlation between IL-22 and Mirt2 was not significant in healthy controls (Figure 2(c)). It is worth noting that analysis of the correlation between IL-22/Mirt2 and plasma C-reactive protein (CRP) revealed that plasma levels of IL-22 (*p* < 0.0001) and Mirt2 (*p* < 0.0001) were significantly and inversely correlated with plasma levels of CRP (data not shown).

### 3.3. Mirt2 Positively Regulated IL-22 in HCnEpCs

**Figure 3:**
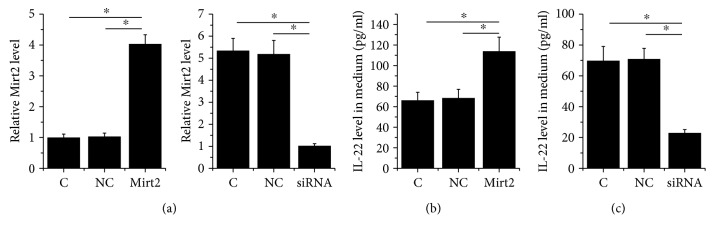
Mirt2 positively regulated IL-22 in HCnEpCs. Mirt2 expression vector or siRNA was transfected into HCnEpCs (treated with LPS as mentioned above). The expression level of Mirt2 was significantly altered in HCnEpCs with Mirt2 expression vector or siRNA transfection at 24 h after transfections comparing C and NC (a). Moreover, Mirt2 overexpression led to increased (b), while Mirt2 siRNA silencing led to decreased secretion of IL-22 from HCnEpCs in medium (c) (^∗^*p* < 0.05).

Mirt2 expression vector or siRNA was transfected into HCnEpCs (treated with LPS as mentioned above). Comparing C and two NC, the expression level of Mirt2 was significantly altered in HCnEpCs with Mirt2 expression vector or siRNA transfection (Figure 3(a), *p* < 0.05), indicating successful transfections. ELISA was performed to measure IL-22 level in medium of different cell groups. Data showed that Mirt2 overexpression led to increased (Figure 3(b), *p* < 0.05), while Mirt2 siRNA silencing led to decreased secretion of IL-22 from HCnEpCs in medium (Figure 3(c), *p* < 0.05).

### 3.4. Mirt2 Inhibited HCnEpC Apoptosis under LPS Treatment

**Figure 4:**
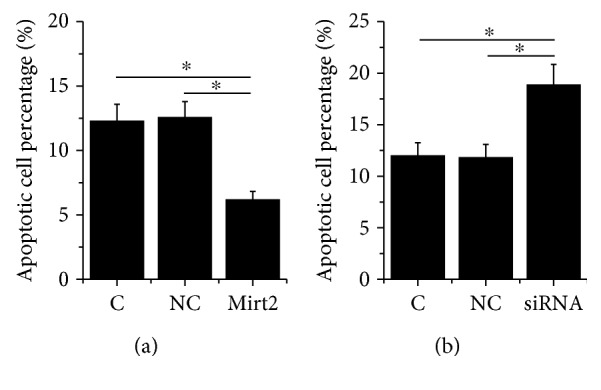
Mirt2 inhibited HCnEpC apoptosis under LPS treatment. Cell apoptosis data analyzed by one-way ANOVA and the Tukey test showed that, comparing C and two NC, Mirt2 overexpression led to decreased (a), while Mirt2 siRNA silencing led to increased (b) apoptotic rate of HCnEpCs (^∗^*p* < 0.05).

Analysis of cell apoptosis assay data showed that, comparing C and two NC, Mirt2 overexpression led to decreased (Figure 4(a), *p* < 0.05), while Mirt2 siRNA silencing led to increased (Figure 4(b), *p* < 0.05) apoptotic rate of HCnEpCs.

## 4. Discussion

The expression pattern, function, and diagnostic values of Mirt2 for UC were analyzed in this paper. We found that Mirt2 was downregulated in UC and Mirt2 may suppress the development of UC by positively regulating anti-inflammatory IL-22.

Different from protein-coding genes, lncRNAs are usually specifically expressed in certain types of cell or during certain developmental stages to participate in specific physiological or pathological processes [12]. However, lncRNAs may be released from the site of synthesis into the blood circulating system to serve as systemic gene expression regulators [13]. In the present study, we detected Mirt2 in plasma of all UC patients and healthy controls, indicating the existence of circulating Mirt2 in the human body. In addition, Mirt2 was downregulated in UC and downregulation of Mirt2 distinguished UC patients from healthy controls. Therefore, circulating Mirt2 may be used to assist the diagnosis of UC.

The anti-inflammatory IL-22 is involved in UC [14]. *Trichuris trichiura* infection has therapeutic effects on UC, and a recent study proved the involvement of IL-22 in this process [15]. In another study, an IL-22-binding protein that can bind to IL-22 to inhibit its protective actions on UC has been characterized [16]. Our study observed the downregulation of IL-22 in UC. In the present study, we found that Mirt2 was a positive regulator of IL-22 in UC because (1) Mirt2 and IL-22 were positively correlated in plasma of UC patients and (2) Mirt2 positively regulated the secretion of IL-22 from HCnEpCs. It is known that Mirt2 can inactivate MAPK pathways to reduce the production of proinflammatory cytokines [11]. IL-22 also has crosstalk with MAPK [17]. Therefore, MAPK may mediate the interaction between IL-22 and Mirt2.

Intestinal epithelial cell apoptosis contributes to the development of UC [18]. In the present study, we showed that Mirt2 has protective effects on HCnEpCs under LPS treatment. Therefore, overexpression of Mirt2 may be a potential approach to treat UC by inhibiting cell apoptosis and promoting the secretion of IL-22. However, more clinical trials are needed.

In conclusion, Mirt2 was downregulated in UC and Mirt2 may suppress the development of UC by positively regulating anti-inflammatory IL-22.

## Data Availability

The analyzed data sets generated during the study are available from the corresponding author on reasonable request.
